# Supplementary material for: Differences between bacteria and eukaryotes in clamp loader mechanism, a conserved process underlying DNA replication
Source: J Biol Chem. 2024 Mar 14;300(4):107166. doi: 10.1016/j.jbc.2024.107166 (PMC11044049; doi:10.1016/j.jbc.2024.107166)
Supplement: Supporting Figure S1 [file mmc1.docx]

**
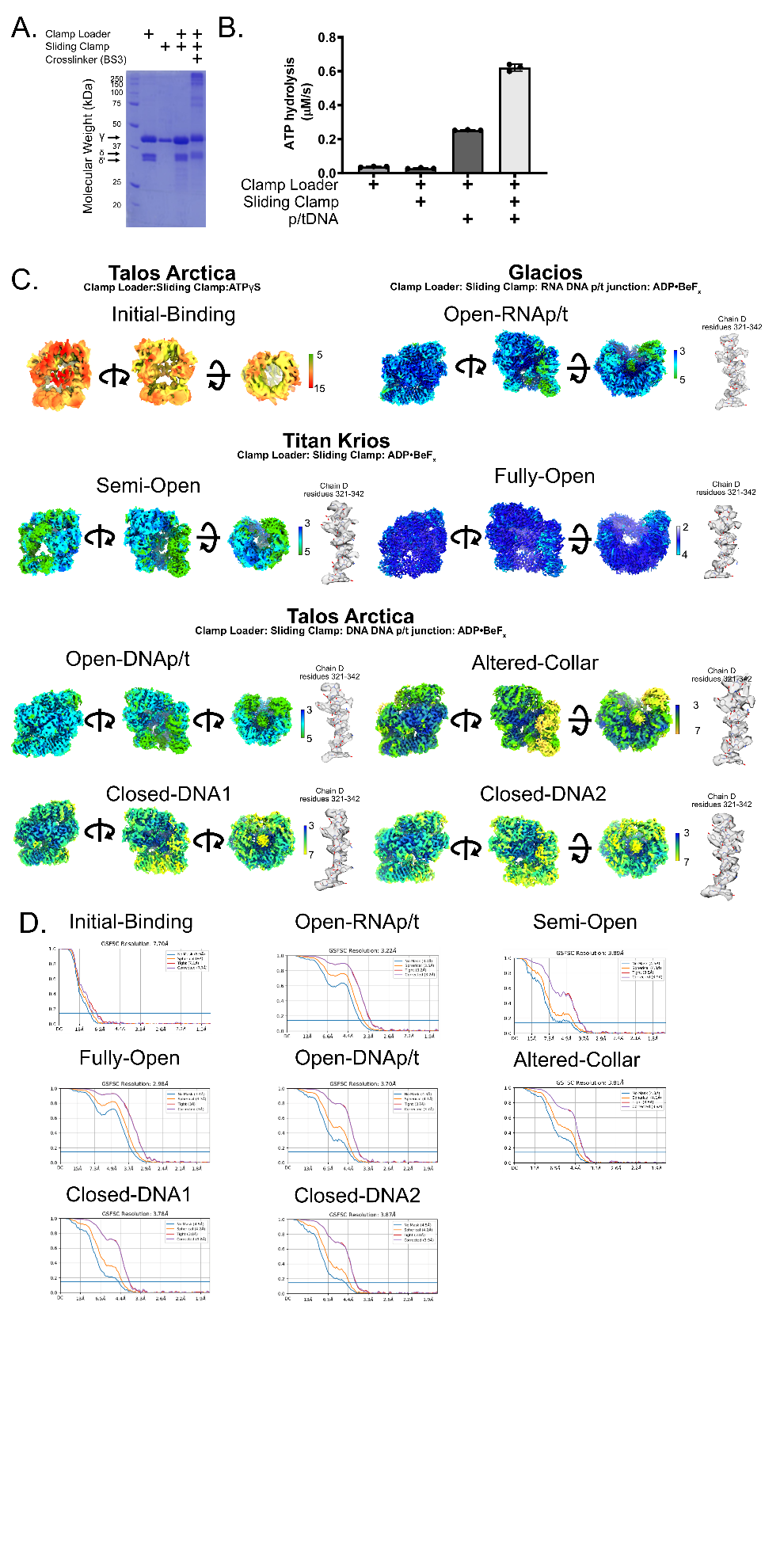
**

**Supplemental Figure 1. Characterization and cryo-EM map validation of the bacterial clamp loader**. **A)** *Purified γ complex and β-clamp*. Sodium dodecyl sulfate-polyacrylamide gel electrophoresis (SDS-PAGE) gel of the purified γ-complex and β-clamp proteins, and samples of the γ complex combined with the β-clamp before and after crosslinking with BS3 crosslinker. **B)** *ATP activity profile of the bacterial clamp loader*. Steady-State ATPase activity of the purified clamp loader complex in combination with the sliding clamp and p/t-DNA (Mean ± S.D., n=3). **C)** *Local resolution and FSC curves of the clamp loader cryo-EM maps*. Local resolution (Fourier shell correlation (FSC)=0.5) of the reconstructions and a representative section of each density map with fitted model. The overall resolution of each map was determined by the FSC of each half-map using Gold-standard cutoff of 0.143 (blue line).
