# Supplementary material for: Differences between bacteria and eukaryotes in clamp loader mechanism, a conserved process underlying DNA replication
Source: J Biol Chem. 2024 Mar 14;300(4):107166. doi: 10.1016/j.jbc.2024.107166 (PMC11044049; doi:10.1016/j.jbc.2024.107166)
Supplement: Supporting Figure S2 [file mmc2.docx]

**
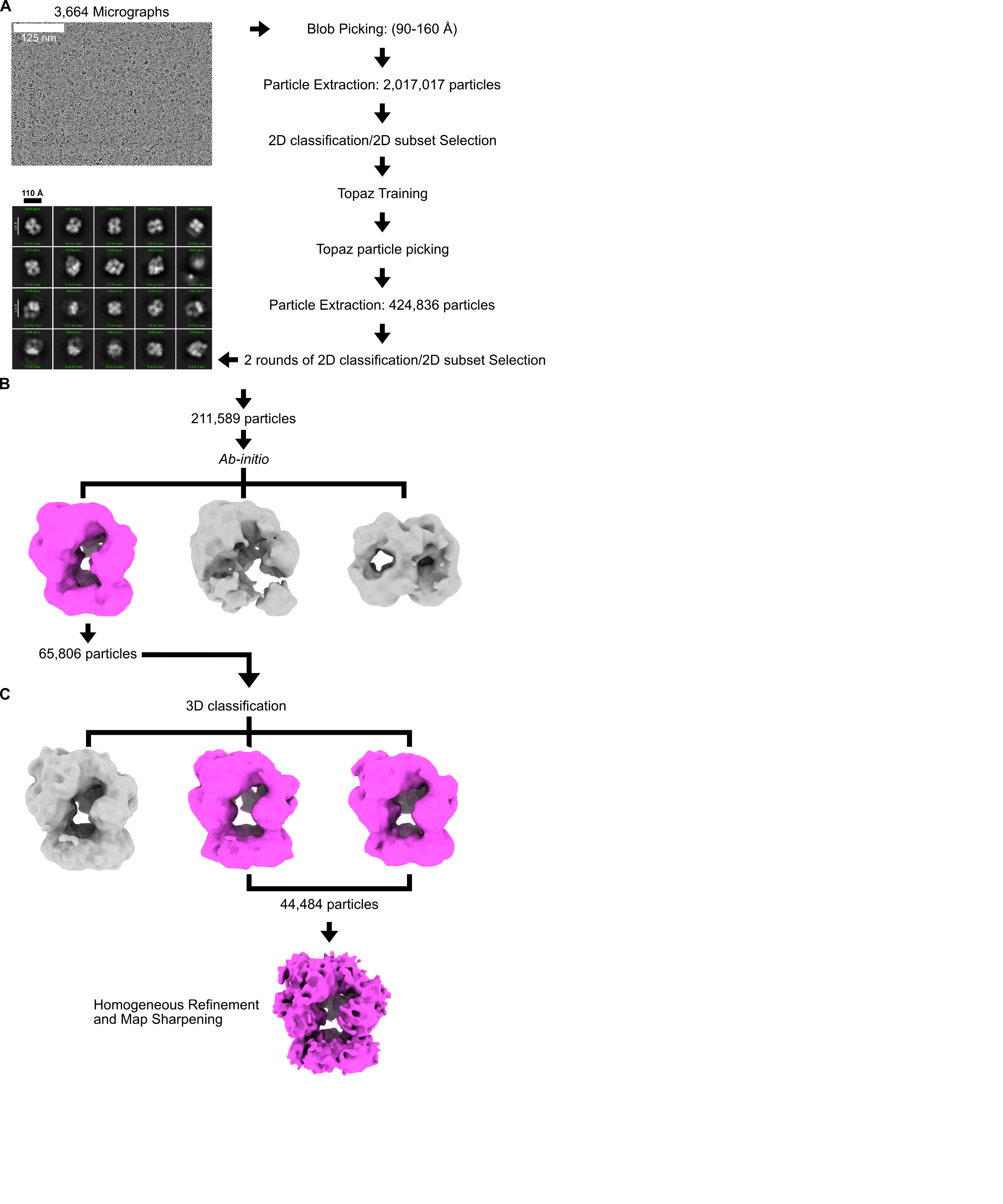
**

**Supplemental Figure 2. Schematic of the Cryo-EM processing workflow for the Clamp Loader, Sliding Clamp, ATPγS dataset.** All data processing was performed using cryoSPARC. **A)** *Representative aligned micrograph and* *particle-picking pipeline*. (Left) Particles were first picked with cryoSPARC’s blob-picker tool. Particles were the extracted and 2D classified. Particles from the selected 2D classes were used as templates to train Topaz. Particles picked by Topaz were then extracted and used for downstream processing. (Right) Representative 2D classes following Topaz particle picking. **B)** *Ab-initio reconstruction.* Particles from the selected 2D classes were used to generate three *Ab-initio* models. One of the three models is of the clamp loader/sliding clamp complex (pink) **C)** *3D classfication and reconstruction*. Particles from the selected *ab-initio* class were 3D classfied into three classes. Two of the 3D classes were combined and homogeneous refinement was used to generate the final 3D reconstruction (pink), which was used to build the Initial-Binding model.
