# Supplementary material for: Differences between bacteria and eukaryotes in clamp loader mechanism, a conserved process underlying DNA replication
Source: J Biol Chem. 2024 Mar 14;300(4):107166. doi: 10.1016/j.jbc.2024.107166 (PMC11044049; doi:10.1016/j.jbc.2024.107166)
Supplement: Supporting Figure S3 [file mmc3.docx]

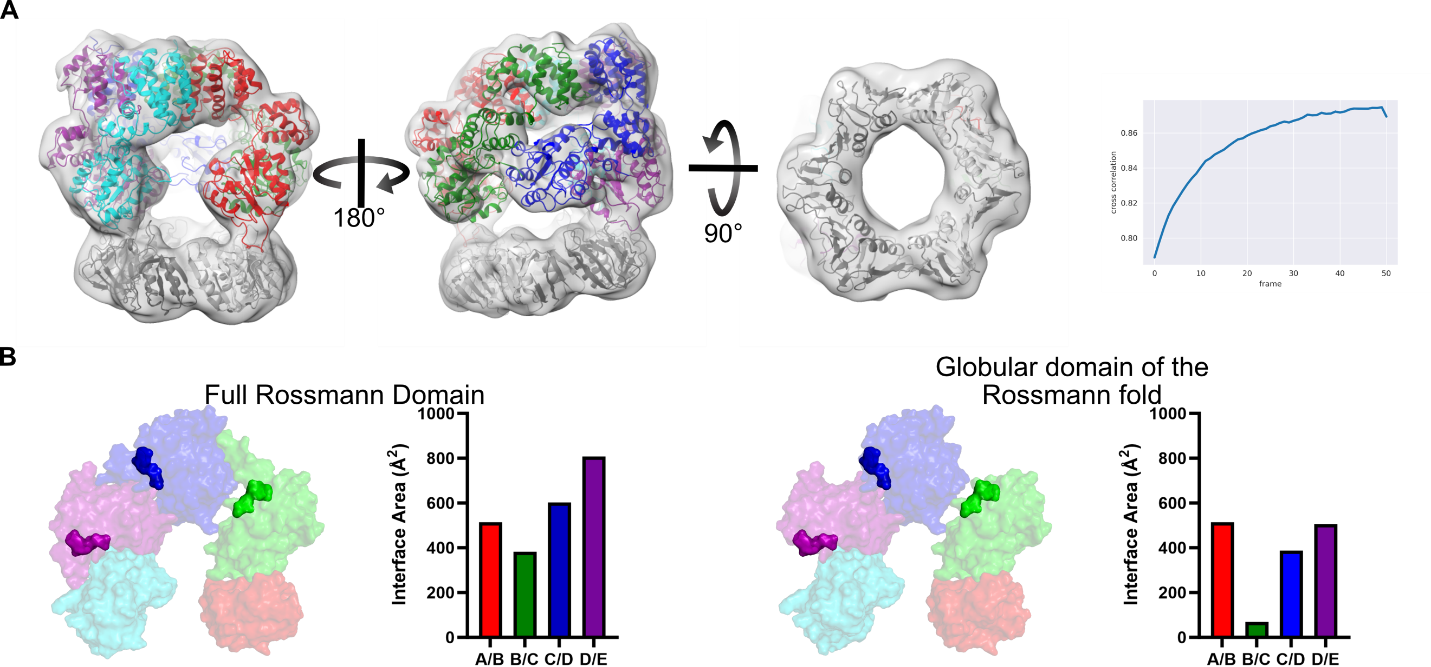


**Supplemental Figure 3. Initial-Binding complex supplemental information. A)** *Molecular Dynamics Flexible Fitting Model of the Initial-Binding complex.* The Initial-Binding complex model is shown fit into the cryo-EM 3D reconstruction. The Model-to-Map cross-correlation throughout the simulation. Energy minimization was applied during the last iteration, which results in a decrease in the overall cross-correlation. **B)** *Geometry of the Rossmann Domain of the Initial-Binding complex.* A transparent surface views of the Rossmann domain of the AAA+ module (left) and globular region of the Rossmann domain (truncating residues 1-10; right) are shown. ATPγS molecules are modelled into the ATP binding sites and shown as solid surfaces. The contact area between adjacent subunits are quantified to the right of each model.
