# Supplementary material for: Differences between bacteria and eukaryotes in clamp loader mechanism, a conserved process underlying DNA replication
Source: J Biol Chem. 2024 Mar 14;300(4):107166. doi: 10.1016/j.jbc.2024.107166 (PMC11044049; doi:10.1016/j.jbc.2024.107166)
Supplement: Supporting Figure S4 [file mmc4.docx]

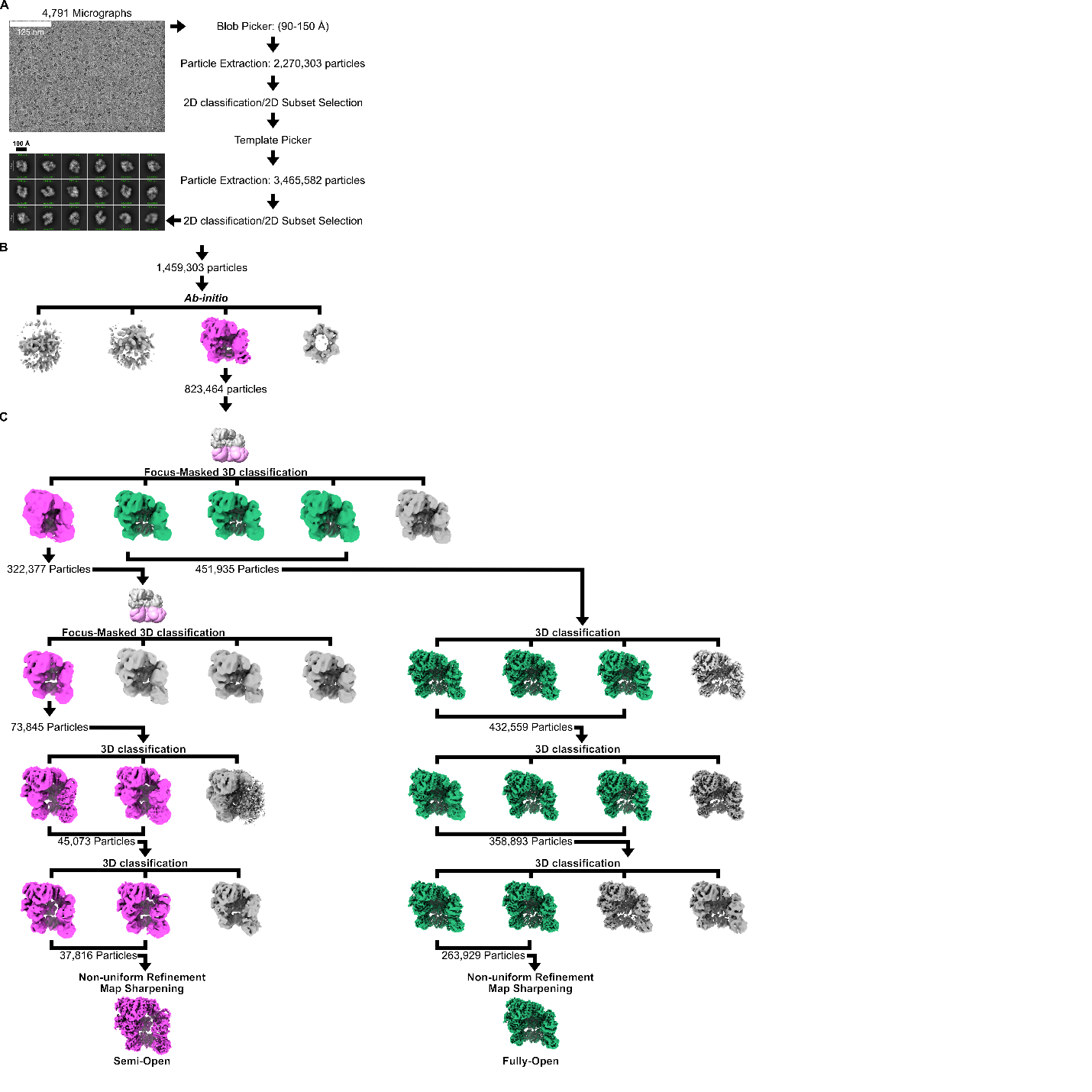


**Supplemental Figure 4. Schematic of the Cryo-EM processing workflow for the Clamp Loader, Sliding Clamp, ADP•BeF_x_ dataset.** All data processing was performed using cryoSPARC. **A)** *A* *representative patch-motion corrected micrograph and particle-picking pipeline.* Particles were first picked with CryoSPARC’s blob-picker tool. Identified particles were extracted and 2D classified. Particles from the selected 2D classes were used as templates for CryoSPARC’s template picker. Particles identified by the template picker were extracted and 2D classified. Representative 2D classes were selected following template picking. **B)** *Ab-initio reconstruction.* Particles from the selected 2D classes were used to generate four *Ab-initio* models. One of the four models resulted in a reconstruction of the clamp loader/sliding clamp complex (pink). **C)** *3D classification and reconstruction.* A focus mask was applied to the sliding clamp of the *ab-initio* model, which was used to perform a focused 3D classiciation of the particles into five classes. One of the resulting classes was in a “semi-open” clamp loader/sliding clamp complex (pink), while three were of the open clamp loader/sliding clamp complex (green). Another focused 3D classification was performed on the on this “Semi-Open” class using a mask on the sliding clamp. One of the resulting classes (pink) was selcted for further 3D classification. Non-uniform refinement and map sharpening was used on the final particle stack. The 3D reconstruction was used to build the Semi-Open model. The three open classes (green, right) were combined and further 3D classified. Non-uniform refinement and map sharpening was used on the final particle stack. The 3D reconstruction was used to build the Fully-Open model.
