# Supplementary material for: Differences between bacteria and eukaryotes in clamp loader mechanism, a conserved process underlying DNA replication
Source: J Biol Chem. 2024 Mar 14;300(4):107166. doi: 10.1016/j.jbc.2024.107166 (PMC11044049; doi:10.1016/j.jbc.2024.107166)
Supplement: Supporting Figure S5 [file mmc5.docx]

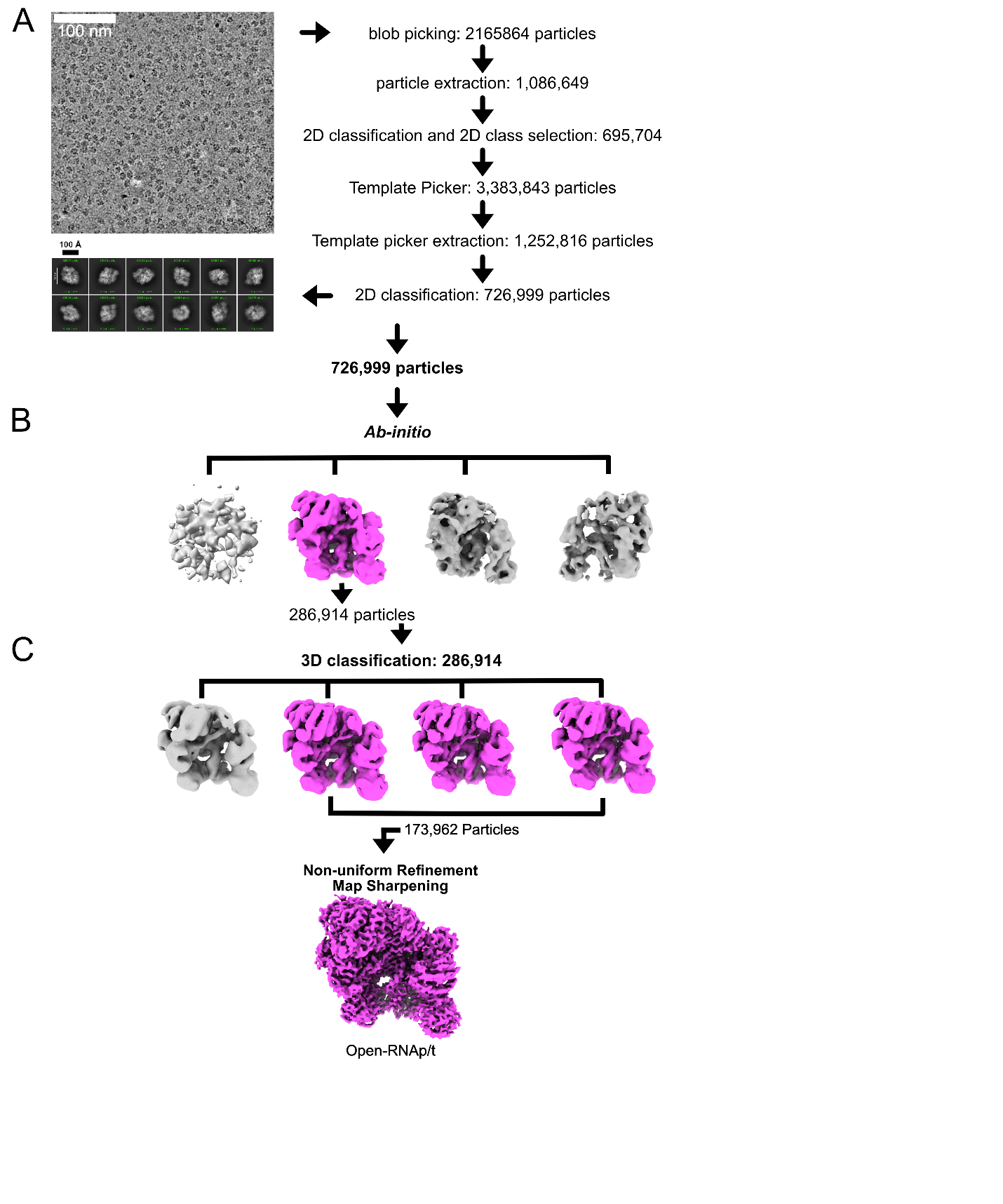


**Supplemental Figure 5. Schematic of the Cryo-EM processing workflow for the Clamp Loader, Sliding Clamp, p/t-RNA, and ADP•BeF_x_ dataset.** All data processing was performed using CryoSPARC. **A)** *A* *representative patch-motion corrected micrograph and particle-picking pipeline.* Particles were first picked with CryoSPARC’s blob-picker tool. Identified particles were extracted and 2D classified. Particles from the selected 2D classes were used as templates for CryoSPARC’s template picker. Particles identified by the template picker were extracted and 2D classified. Representative 2D classes were selected following template picking. **B)** *Ab-initio reconstruction.* Particles from the selected 2D classes were used to generate four *Ab-initio* models. One of the four models resulted in a reconstruction of the clamp loader/sliding clamp/p/t-RNA complex (pink). **C)** *3D classification and reconstruction.*  The selected particles were 3D classified into four classes. Non-uniform refinement and map sharpening was used on the final particle stack. This 3D recontruction was used to build the Open-RNAp/t model.
