# Supplementary material for: Differences between bacteria and eukaryotes in clamp loader mechanism, a conserved process underlying DNA replication
Source: J Biol Chem. 2024 Mar 14;300(4):107166. doi: 10.1016/j.jbc.2024.107166 (PMC11044049; doi:10.1016/j.jbc.2024.107166)
Supplement: Supporting Figure S6 [file mmc6.docx]

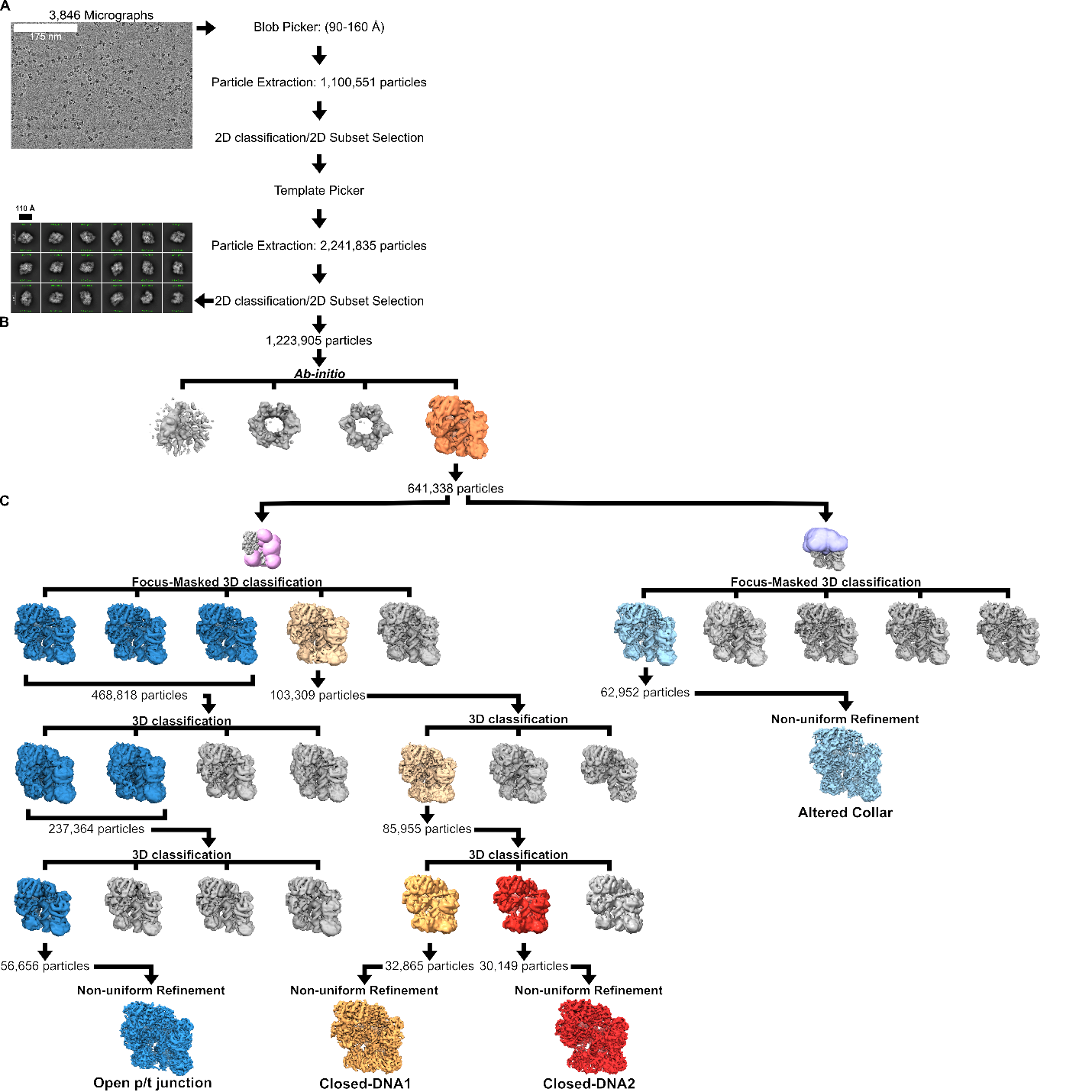
**Supplemental Figure 6.** **Schematic of the Cryo-EM processing workflow for the Clamp Loader, Sliding Clamp, p/t-DNA, and ADP•BeF_x_ dataset.** All data processing was performed using CryoSPARC. **A)** *A* *representative patch-motion corrected micrograph and particle-picking pipeline*. Particles were first picked with CryoSPARC’s blob-picker tool, extracted, and 2D classified. Particles from the selected 2D classes were used as templates for CryoSPARC’s template picker. Particles identified by the template picker were extracted and 2D classified. **B)** *Ab-initio reconstruction.* Particles from the selected 2D classes were used to generate four *Ab-initio* models. One of the four models consisted of the clamp loader/sliding clamp/p/t-DNA complex (orange) **C)** *3D classification and reconstruction.* The first round of 3D classification was performed by applying a focus mask to the sliding clamp and the A and B subunits of the clamp loader to generate five classes. This resulted in three open clamp loader/sliding clamp/p/t-DNA complexes (blue) and one closed clamp loader/sliding clamp complex (tan). The three clamp loader/sliding clamp/p/t-DNA complexes were pooled and iteratively 3D classified. The final class had density for the clamp loader, p/t-DNA, and all six domains of the sliding clamp. The closed class was 3D classified resulting in two classes of the closed clamp loader/sliding clamp/DNAp/t complex (orange and red). After 3D classification, non-uniform refinement was performed to generate the final reconstructions of the Open-p/t-DNA, Closed-DNA1, and Closed-DNA2 complexes. In parallel, the particles from the *ab-initio* model were 3D classified into five classes while appling a focus mask to the collar and lid domains of the clamp loader/sliding clamp/DNAp/t complex (right). One of the classes had an altered collar (cyan). After 3D classification, non-uniform refinement was performed to generate the final reconstruction of the Altered-Collar class.
