# Supplementary material for: Differences between bacteria and eukaryotes in clamp loader mechanism, a conserved process underlying DNA replication
Source: J Biol Chem. 2024 Mar 14;300(4):107166. doi: 10.1016/j.jbc.2024.107166 (PMC11044049; doi:10.1016/j.jbc.2024.107166)
Supplement: Supporting Figure S7 [file mmc7.docx]

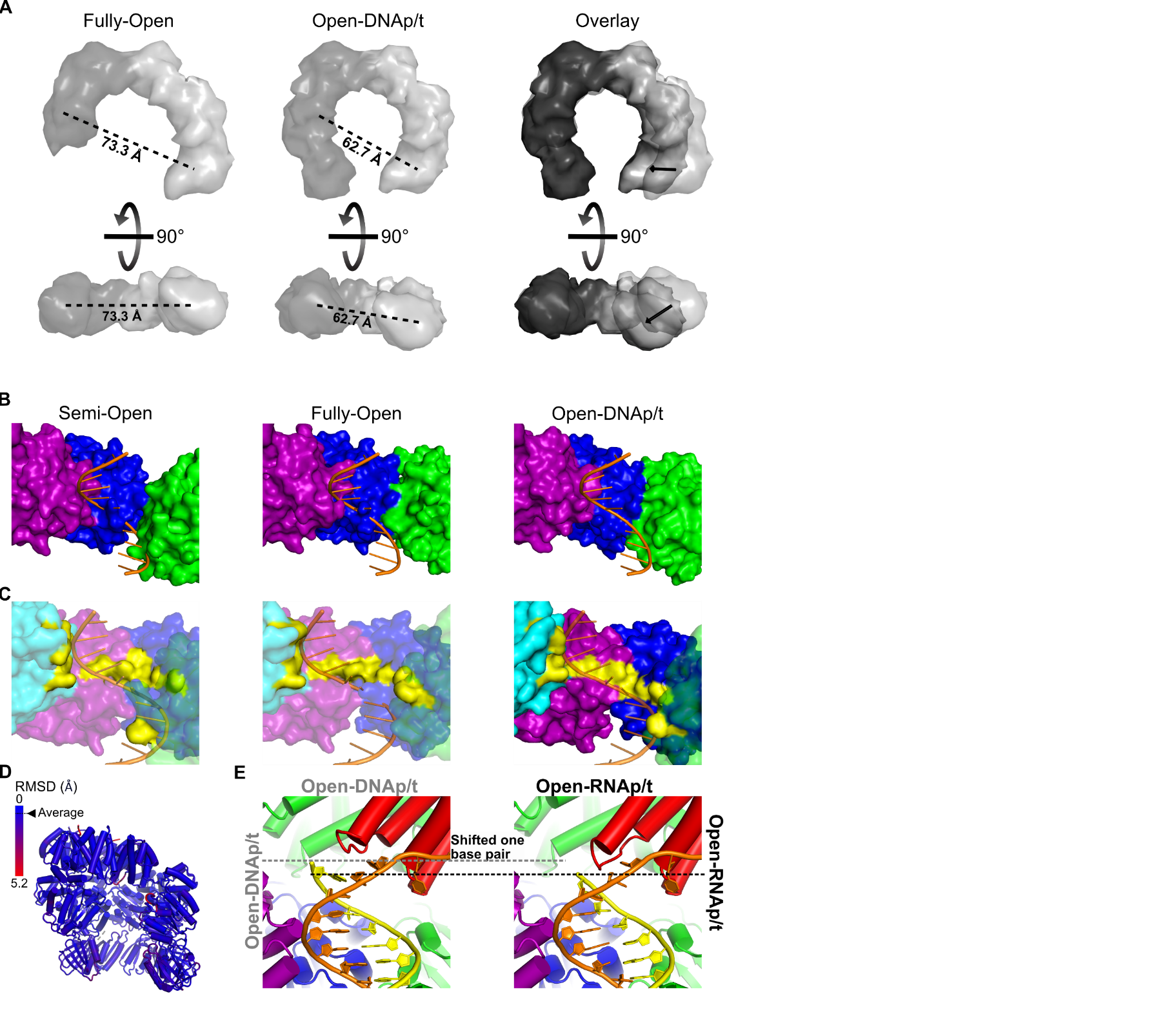


**Supplemental Figure 7. Conformational change induced by p/t-junction binding. A)** *Conformational changes of the sliding clamp between states.* Models of the sliding clamp of the Fully-Open and Open-DNAp/t states are displayed as low contour surfaces. The distance between domain III of subunit I and domain II of subunit of II of the sliding clamp in each state is shown. The Fully-Open and Open-DNAp/t sliding clamps were aligned on subunit II and displacements of domains within subunit I are shown as vectors. **B)** *Compatibility of the Rossmann domain with template DNA binding*. Surfaces of the Rossmann domains of the B-D subunits of the Semi-Open, Fully-Open, and Open-DNAp/t models are shown. Models were aligned on the D subunit and the template strand from the Open-DNAp/t model is superimposed on the Semi-Open and Fully-Open models. **C)** *Compatibility of template binding residues with template DNA binding*. Surfaces of the Rossmann domains of the B-E subunits of the Semi-Open, Fully-Open, and Open-DNAp/t models are shown. Models were aligned on the E subunit and the template strand from the Open-DNAp/t model is superimposed on the Semi-Open and Fully-Open models. Key template strand gripping residues are highlighted in yellow. **D)** Per residue RMSD of the clamp loader/sliding clamp complex comparing the Open DNAp/t and Open-RNAp/t states. Structures were globally aligned and per-residue C_α_ RMSD was calculated. **E)** *Alignment of template DNA binding residues with the template strand in different states*. Surfaces are shown of the Rossmann domains of the B-E subunits of the Semi-Open, Fully-Open, and Open-DNAp/t models. Models were aligned on the E subunit and the template strand from the Open-DNAp/t model is superimposed on the Semi-Open and Fully-Open models. Residues that bind to the template strand are shown in yellow.
